# Mechanistic modeling of pesticide uptake with a 3D plant architecture model

**DOI:** 10.1007/s11356-021-14878-3

**Published:** 2021-06-17

**Authors:** Helena Jorda, Katrin Huber, Asta Kunkel, Jan Vanderborght, Mathieu Javaux, Christoph Oberdörster, Klaus Hammel, Andrea Schnepf

**Affiliations:** 1 Institute of Bio- and Geosciences, Agrosphere Institute, IBG-3, Forschungszentrum Jülich GmbH, Wilhelm-Johnen-Strasse, 52428 Jülich, Germany; 2 Department of Earth and Environmental Sciences, Faculty of Bioscience Engineering, KU Leuven, Kasteelpark Arenberg 20, 3001 Leuven, Belgium; 3 Earth and Life Institute-Environnemental Sciences, Université Catholique de Louvain, Croix du Sud 2, 1348 Louvain-la-Neuve, Belgium; 4 Research & Development, Crop Science, Bayer AG, Alfred-Nobel-Str 50, 40789 Monheim am Rhein, Germany

**Keywords:** Mechanistic pesticide uptake model, 3-D root architectural model, Advective uptake, Diffusive uptake, PEARL model, FOCUS scenarios

## Abstract

Meaningful assessment of pesticide fate in soils and plants is based on fate models that represent all relevant processes. With mechanistic models, these processes can be simulated based on soil, substance, and plant properties. We present a mechanistic model that simulates pesticide uptake from soil and investigate how it is influenced, depending on the governing uptake process, by root and substance properties and by distributions of the substance and water in the soil profile. A new root solute uptake model based on a lumped version of the Trapp model (Trapp, 2000) was implemented in a coupled version of R-SWMS-ParTrace models for 3-D water flow and solute transport in soil and root systems. Solute uptake was modeled as two individual processes: advection with the transpiration stream and diffusion through the root membrane. We set up the model for a FOCUS scenario used in the European Union (EU) for pesticide registration. Considering a single vertical root and advective uptake only, the root hydraulic properties could be defined so that water and substance uptake and substance fate in soil showed a good agreement with the results of the 1D PEARL model, one of the reference models used in the EU for pesticide registration. Simulations with a complex root system and using root hydraulic parameters reported in the literature predicted larger water uptake from the upper root zone, leading to larger pesticide uptake when pesticides are concentrated in the upper root zone. Dilution of root water concentrations at the top root zone with water with low pesticide concentration taken up from the bottom of the root zone leads to larger uptake of solute when uptake was simulated as a diffusive process. This illustrates the importance of modeling uptake mechanistically and considering root and solute physical and chemical properties, especially when root-zone pesticide concentrations are non-uniform.

**Supplementary Information:**

The online version contains supplementary material available at 10.1007/s11356-021-14878-3.

## Introduction

Leaching of plant protection products and their metabolites into the groundwater presents a major risk to the environment and to public health. Maximum residue limits for pesticides in groundwater, drinking water, and food products are set at international and national levels (Li and Jennings, 2017). In addition, different risk assessment studies are required when new products are placed into markets. In the European Union (EU), such assessments are based on modeling, laboratory, and field studies (EU Parliament, 2009).

A framework by the FOCUS (forum for the coordination of pesticide fate models and their use) group provides four 1D models (e.g., PEARL model (Leistra et al., 2000)) and standard model scenarios, which are used to assess the risk for pesticide enrichment in ground and surface water (Boesten et al., 2000). These models describe all relevant processes that determine the fate of pesticides in soils, including the uptake of pesticides by plant roots. All four EU models describe pesticide uptake by plants as a passive advective process (see Table 1), i.e., pesticides are taken up proportionally to water uptake flux. The solute fraction that is taken up is determined by a constant factor, defined as the plant uptake factor (PUF) or the transpiration stream concentration factor (TSCF):

1 $$ {R}_u={R}_{u,L}\ast \left[ TSCF\ or\  PUF\right]\ast {C}_{S,l}, $$

**Table 1:** Different root solute uptake mechanisms. J_U_ is the solute uptake flux [M L^-2^ T^-1^], ε is the advection solute uptake fraction [-], J_w,r_ is the water uptake flux (which is defined positive when the root takes up water) [L T^-1^], C_S,l_ is the dissolved soil solute concentration [M L^-3^], D_cell_ is the cell membrane diffusion coefficient [T^-1^], Δx is diffusion length [L], C_R,l_ is the dissolved root solute concentration [M L^-3^], V_max_ is the maximum rate [M L^-2^ T^-1^], and K_m_ is the Michaelis constant [M L^-3^]

Mechanism	Solute flux [M L^-2^ T^-1^]	Example
Exclusion	*J*_*U*_ = 0	Salt
Passive – advection	*J*_*U*_ = *εJ*_*w*, *r*_*C*_*S*, *l*_	Nutrients, organic solutes (pesticides)
Passive - diffusion	${J}_U=\frac{D_{cell}}{\Delta x}\left({C}_{S,l}-{C}_{R,l}\right)$	Organic solutes (pesticides)
Active uptake (e.g., Michaelis-Menten)	${J}_U=\frac{V_{max}{C}_{S,l}}{K_m+{C}_{S,l}}$	Nutrients, ions

where *R*_*u*_ [M L^-3^ T^-1^] is the mass rate of pesticide uptake per bulk volume of soil, *R*_*u,L*_ [L^3^ L^-3^ T^-1^] the water uptake rate, and *C*_*S,l*_ [M L^-3^] the dissolved solute concentration in the soil.

The PUF can be calculated as:

2 $$ PUF\left({t}_1\right)=\frac{\sum_{t_0}^{t_1}{m}_{upt}/{T}_{act}}{\overline{C_{S,l}}\left({t}_1\right)} $$

where m_upt_ [M] is the mass taken up by the root system within one day, *T*_*act*_ [L^3^] is the volume of water transpired within this day and $\overline{C_{S,l}}$ is the mean dissolved soil solute concentration in the root zone [M L^-3^].

The TSCF is calculated as:

3 $$ TSCF\ \left({t}_1\right)=\frac{\sum_{t_0}^{t_1}{m}_{collar}/{T}_{act}}{\overline{C_{S,l}}\left({t}_1\right)} $$

where *m*_*collar*_ [M] is the mass which is moved within one day from the root system to the above ground plant.

The appropriateness and exchangeability of PUF and TSCF to model solute root uptake has been discussed within the FOCUS developer group (Boesten et al., 2014). In an earlier FOCUS report (Boesten et al., 2000), a plant uptake factor between 0 and 1 was used. For tier 1 assessment, the factor was set to 0.5, or for non-systemic compounds to zero. In higher tier assessment, the modeler can adapt the uptake factor according to experimental data.

In more general terms, solute uptake by roots can be described by four main mechanisms: active uptake, advective passive uptake, diffusive passive uptake, and solute exclusion (Table 1). Although a few herbicides have been reported to be taken up actively (Sterling, 1994), most pesticides are taken up by roots via advective and/or diffusive passive uptake pathways. Advective passive uptake represents the uptake of solute together with the water flow into the roots, whereas diffusive passive uptake is caused by a concentration gradient between root and soil. For pesticides, it has been discussed that the ratio of advective to diffusive passive uptake and their translocation from root to shoot are largely controlled by the compound’s polarity and lipophilic properties (Briggs et al., 1982; Sicbaldi et al., 1997; Trapp, 2000). Highly lipophilic pesticides that are in solution can dissolve into the cell membrane’s phospholipid bilayer and reenter the aqueous phase at the other side, causing their uptake to be dominated by diffusive mechanisms, which can occur by roots that are not necessarily active in taking up water (Sicbaldi et al., 1997). In addition, transport of pesticides from the root surface to the xylem vessels can occur via the apoplastic or the symplastic pathway. More lipophilic compounds tend to take the symplastic pathway by crossing the cell membranes whereas less lipophilic substances travel the apoplastic route through the cell wall space. The ratio between these two routes is highly dominated by the compound’s lipophilicity, and it determines the overall transport to the shoot (Sicbaldi et al., 1997). Larger transport of pesticide from root to shoot has been observed for moderately lipophilic compounds (Briggs et al., 1982; Collins et al., 2006; Hsu et al., 1990; Sicbaldi et al., 1997). However, the mechanistic understanding of pesticide uptake and translocation via the root system remains challenging (Collins et al., 2006).

To accurately calculate pesticide uptake according to these mechanisms (Table 1), models need to explicitly account for pesticide concentrations around (C_S,l_) and within (C_R,l_) roots and water fluxes at the root surfaces (J_w,r_). This means that such a model would require (a) to represent the 3-dimensional root system to access these local pesticide concentrations around roots and (b) to account for the transport and fate of pesticide within the root system.

Most current models that simulate pesticide uptake and transport in plants consider these different processes only partly. Pesticide-fate models such as PEARL represent solute uptake by the root as advective uptake only and do not consider diffusive uptake that depends on concentration gradients between root and soil. Only solute concentrations within the soil domain and root water uptake are simulated and solute transport inside the plant is not considered. DynamiCROP (Fantke et al., 2011), a model developed for the assessment of pesticide exposure to humans and uptake by plants, considers diffusive uptake of pesticide by the root, but since it is not spatially resolved, it does not consider spatial distribution of pesticides in the root zone. Kim et al. (2004) developed a radially symmetric single root model describing the transport of TNT (2,4,6-trinitrotoluene) from bulk soil to the root surface and its uptake by the root. They described solute uptake from the soil to the root by an uptake rate obtained from experimental data. The net translocation of solute from soil to leaf was simulated as an advective process based on transpiration rate, solute concentrations at the root surface, and a TSCF determined by experimental data. Trapp (2000) developed a model of solute uptake suitable for non-ionic and ionic compounds that considers flow between different root compartments, cortex, steel, and xylem vessels and allows for both diffusive and advective uptake from the soil. Recently, Brunetti et al. (2019) coupled the HYDRUS model with the Trapp model (Trapp, 2007) for modeling the transport and transformation of solutes in the soil-plant continuum. Although their model accounts for advective solute uptake with the transpiration stream and solute translocation to the shoot, it does not consider diffusive uptake across the root membrane nor does it simulate 3D explicit root architecture and water flow from the soil to the root system and within the root system. Duncan et al. (2019) developed a mathematical model that accounts for the impact of water and solute uptake on tuber growth. However, 3D root architecture is also not considered.

Three-dimensional solute and water uptake models exist (Schröder et al., 2013; Šimůnek and Hopmans, 2009) but none of these is able to explicitly represent all uptake mechanisms. Therefore little is known about the spatial distribution of pesticide uptake and how it affects pesticide fate in plants and soils. As existing models for root uptake of organic solutes are either lacking a mechanistic description of uptake, are single root (radial-symmetric) models, or are 1D models, our aim was to create a model capable to model solute uptake from 3D soil that considers 3D hydraulic root architecture, pesticide transport in soil and roots as well as both advective and diffusive uptake mechanisms. In particular, we extended the existing 3D R-SWMS model (Javaux et al., 2008), simulating water movement and solute transport within the soil and root system (Bechtold et al., 2011; Huber et al., 2014; Schröder et al., 2013), to include mechanistic root solute uptake according to Trapp (2000). We then tested the model using a setup extracted from the FOCUS scenarios and compared simulation results with those obtained by the PEARL model.

## Theory

### Water model

Water fluxes in the 3D soil, into and in the 3D root architecture were simulated using the R-SWMS model (Javaux et al., 2008). Water flow in the soil is obtained by solving the Richards equation. Furthermore, the root system is represented by a network of connected segments (i.e., root system architecture), characterized by their length, radius, and (xylem and radial) hydraulic properties. Water uptake by each root segment is obtained by solving a Darcy-like equation that describes radial flow across the root surface, J_w,r_ [L T^-1^], as

4 $$ {J}_{w,r}={L}_r\left[{h}_S-{h}_x\right] $$

where L_r_ [L L^-1^ T^-1^] is the radial conductivity, and h_S_ and h_x_ [L] are the water potentials at the root surface and xylem, respectively.

The water sink, S_w_ [T^-1^], in a soil element is then calculated as

5 $$ {S}_w=\frac{\sum_{i=1}^n{q}_{r,i}}{V_S} $$

where q_r,i_ [L^3^ T^-1^] is the radial flow in segment i, n is the number of root segments in a soil element, and V_S_ [L^3^] is the volume of a soil element.

Xylem water potential in the root is obtained by solving the linear equation of water flow in the root (Doussan et al., 1998)

6 $$ {J}_{w,x}=-\frac{K_x}{A_x}\left(\frac{\Delta  {h}_x}{l}+\frac{\Delta  z}{l}\right) $$

where J_w,x_ [L T^-1^] is the axial water flux within the root, K_x_ [L^4^ L^-1^ T^-1^] is the axial conductance, A_x_ [L^2^] is the cross-section area of the segment, and l [L] is the root segment length.

Both the root segment’s radial conductivity and axial conductance can change with age and root segment type. We refer to Javaux et al. (2008) for a detailed description of this section.

### Solute model

Solute transport and fate in the soil was simulated using a particle tracker algorithm, ParTrace (Bechtold et al., 2011), which solves convective and diffusive transport, degradation, and sorption. The solute conservation equation in the soil is described by

7 $$ \frac{\partial \left({\theta}_S+{\rho}_b{K}_{D,S}\right){C}_{S,l}}{\partial t}=\nabla \cdotp \left( D\tau {\theta}_S\nabla {C}_{S,l}-{\mathrm{J}}_{\mathrm{w},\mathrm{S}}{C}_{S,l}\right)-\left({\theta}_S+{\rho}_b{K}_{D,S}\right){k}_S{C}_{S,l}+{S}_S $$

where *θ*_*S*_ [-] is the soil water content, *ρ*_*b*_ [M L^-3^] the dry soil bulk density, *K*_*D,S*_ [L^3^ M^-1^] the solute partitioning coefficient between the sorbed and dissolved phases in the soil, *D* [L^2^ T^-1^] the soil diffusion coefficient in water, *τ* [-] the impedance factor, *J*_*w,S*_ [L T^-1^] the soil water flux, *k*_*S*_ [T^-1^] the first-order degradation rate constant, and *S*_*S*_ [M L^-3^ T^-1^] the solute sink for root uptake in the soil.

Solute degradation acts on both dissolved and adsorbed solutes equally and is described as a first order process depending on soil moisture, temperature, and soil depth (Leistra et al., 2000). Further information on degradation can be found in the Supporting Information (SI).

**Fig. 1:**
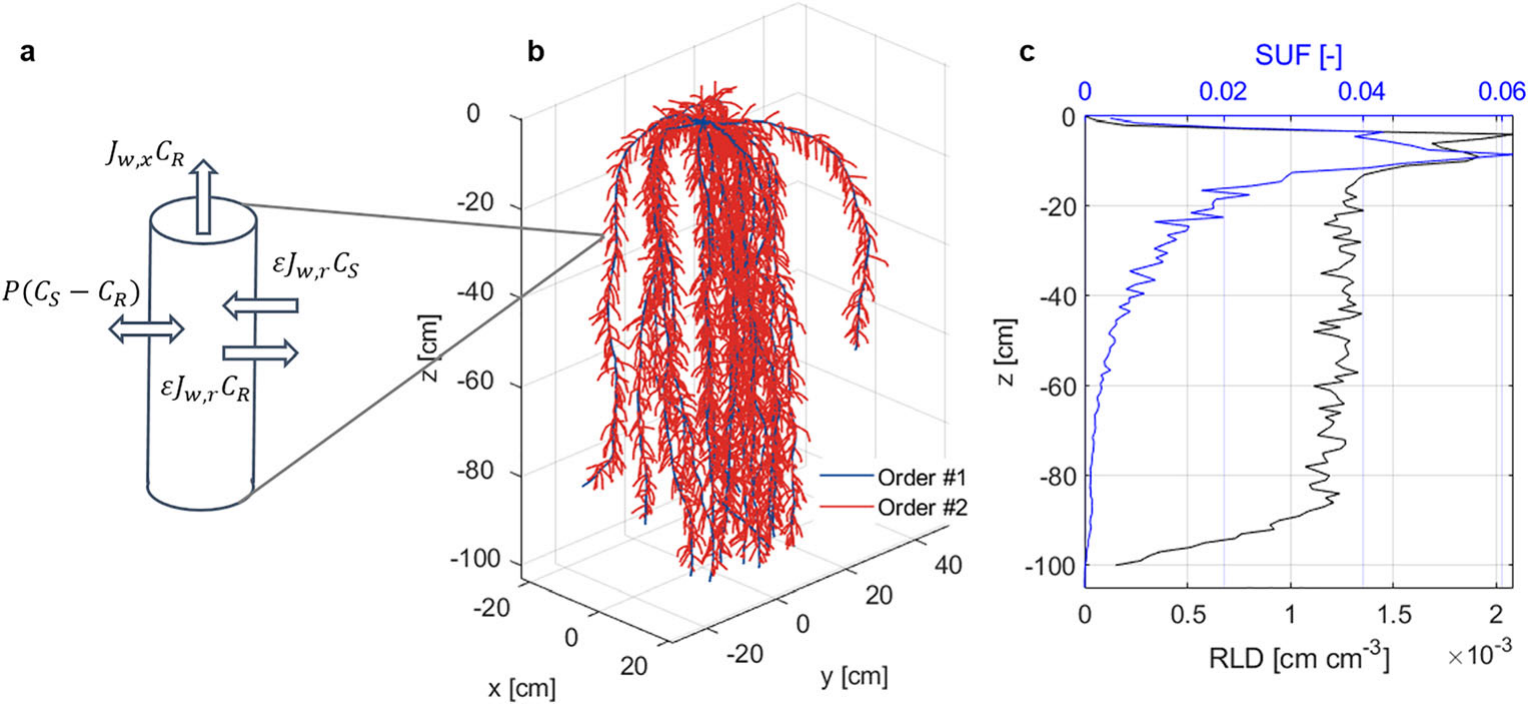
**a** Conceptual model of radial and axial solute fluxes into an individual root segment, consisting of one lumped root compartment, enclosed by a membrane. P [L T^-1^] is the membrane permeability, J_w,r_ [L T^-1^] is the radial root water uptake flux, C_S_ [M L^-3^] the dissolved solute concentration in the surrounding soil environment, and C_R_ [M L^-3^] dissolved root concentration. ε [-] denotes the proportion of solute which can enter the root via convection and J_w,x_ [L T^-1^] the axial water flux. Solute fluxes are calculated for each segment that constitutes the 3D root system. 3D representation of the simulated maize root system used in this study (**b**) and its depth profiles of root length density and standard uptake fraction (SUF) (**c**) at its full development

In our representation of pesticide uptake, we followed the approach of Trapp (2000) for neutral compounds. However, we simplified the root such that it is represented by a single compartment (Fig. 1a), whereas Trapp (2000) differentiates between root cortex and the central cylinder. Two passive uptake pathways exist for root solute uptake: diffusion through cell membranes and advective uptake with the root water uptake flux through the apoplast. Solute uptake via the apoplast is possible due imperfections of the root’s Casparian bands (Schreiber et al., 1999). The Casparian bands are made out of lipophilic and aromatic substances that are thought to block the transport of water and solutes through the apoplast. However, there is evidence that these barriers are not flawless (Freundl et al., 1998). Solutes can also diffuse across the lipid phase of the membrane as a function of the concentration gradient between soil and root and a diffusion coefficient, which is represented as root permeability. Perfect mixing of the solute within the root is assumed. While in Trapp (2000), a full root system is represented by a defined volume and surface area, we apply the model to each single root segment.

To enter the root the solute has to pass cell walls and cell membranes, whose overall root permeability, P [L T^-1^], can be obtained by

8 $$ \mathrm{P}=\frac{1}{\raisebox{1ex}{$1$}\!\left/ \!\raisebox{-1ex}{${P}_W$}\right.+\raisebox{1ex}{$1$}\!\left/ \!\raisebox{-1ex}{${P}_M$}\right.}, $$

where the cell wall permeability is P_W_ = 2.5 × 10^-4^ m s^-1^. The membrane permeability P_M_ depends on the solute lipophilic properties and several experimental relations exist describing this dependency. The following relationship was proposed by Trapp (2000) for neutral compounds based on results from Grayson and Kleier (1990) and Hsu and Kleier (1996):

9 $$ \mathit{\log}{P}_M=1.20\ \mathit{\log}{K}_{OW}-7.50 $$

where P_M_ is expressed in m s^-1^ and K_OW_ is the solute octanol water partitioning coefficient.

Once entered the root, the solute can be sorbed to plant tissue or dissolved in the free root water. The sorbed portion of the solutes does not take part in transport processes. Since a dynamic equilibrium between sorbed and desorbed fractions is assumed, sorption does not correspond with an irreversible immobilization but with a retardation of transport processes. The solved solute can move with the axial root water flow and leave the root system (to the above ground plant) or can be released back into the soil, due to changing gradient or water flow conditions.

Assuming that the solute diffusion within the root is equal to that of water, with a diffusion coefficient *D* of approximately 1 cm^2^ d^-1^ (Hayduk and Laudie, 1974), an approximate velocity *v* of convective flow of 1000 cm d^-1^ for mature plants, and an average root diameter *d* of 0.1 cm results in a Péclet number (*=dvD*^*-1*^) of 100. Thus, solute transport within the root is dominated by convection and diffusive transport is neglected. The solute conservation equation within the root is thus given by

10 $$ \left({\theta}_R+{\rho}_R{K}_{D,R}\right)\frac{\partial {C}_{R,l}}{\partial t}=\nabla \cdotp \left(-{\mathrm{J}}_{\mathrm{w},\mathrm{x}}{C}_{R,l}\right)-\left({\theta}_R+{\rho}_R{K}_{D,R}\right){k}_R{C}_{R,l}+{S}_R, $$

where *θ*_*R*_ [-] is the root water content, *ρ*_*R*_ [M L^-3^] the dry root bulk density, *K*_*D,R*_ [L^3^ M^-1^] the partitioning coefficient in the root, *C*_*R,l*_ [M L^-3^] the dissolved solute concentration in the root, *k*_*R*_ [T^-1^] the degradation constant, and *S*_*R*_ [M L^-3^ T^-1^] the solute source term.

Following Trapp (2000), we described the source term for solute uptake into each root segment by

11 $$ {S}_R=\frac{A_R}{V_R}\left(P\left({C}_{S,l}-{C}_{R,l}\right)+\varepsilon {J}_{w,r}{C}_{S,l}\right), $$

where *A*_*R*_ [L^2^] is the outer root surface of the segment (without root hairs), *V*_*R*_ [L^3^] the root segment’s volume, and *ε* [-] the ratio of the actual convective uptake to the convective uptake if the solute could freely flow with the water into the root. *ε* can also be understood as 1-σ, where σ is the reflection coefficient that refers to the ability of the membrane to prevent passive transport of solutes through the apoplast (Hopmans and Bristow, 2002).

The sink term for solute uptake from soil is equal to the sum of radial flow of solutes over all root segments, *i*, within one soil element *V*_*S*_

12 $$ {S}_S=-\frac{\sum_{i=1}^n{A}_{R,i}\left(P\left({C}_{S,l}-{C}_{R,l,i}\right)+\varepsilon {J}_{w,i}{C}_{S,l}\right)}{V_S} $$

A detailed explanation of the numerical implementation of the solute uptake model supported by a convergence study can be found in SI.

## Materials and methods

The model PEARL is one-dimensional and considers only vertical distributions, flow, and transport of water and pesticides (Leistra et al., 2000). The model has been validated against experimental datasets (Vanclooster et al., 2000; 2003) and it represents the relevant processes that determine pesticide fate in soils and pesticide leaching. R-SWMS, on the other hand, is a three-dimensional model where the soil as well as the root domains are spatially resolved (Javaux et al., 2008). The Hamburg FOCUS scenarios was chosen and adapted for 3D simulations with the coupled version of R-SWMS and ParTrace models. We directly applied the prescribed model parameters from the FOCUS scenario or derived new parameters for R-SWMS. This way we compared the 3D model results to the 1D solution which is used in European pesticide registration.

The FOCUS scenarios use a 6-year warm up period followed by a 20-year simulation time. As such a long simulation period is not feasible in R-SWMS due to computational demand, we chose to simulate one growing season. We used the PEARL model output to generate the initial conditions in the soil domain, i.e., hydraulic head and solute concentration distribution. Hereby, we assumed horizontally homogeneous initial water and solute states at the beginning of the one growing season simulation.

### Maize as crop

We chose maize as crop because the root system architecture is well known and it is a summer crop, which allows us to explore the influences of water availability on pesticide fate. Although the maximum simulated rooting depth for maize in the FOCUS scenario is −100 cm, a soil domain with a depth of −145 cm was chosen for the R-SWMS simulations. Extending the soil domain beyond the rooting zone was necessary in order to avoid boundary effects. From PEARL simulations, we observed a relatively constant solute concentration at −145 cm depth, which we set as boundary condition (BC) for R-SWMS such that it could consider advective transport of solute from deeper soil layers to the root zone.

### Boundary conditions

The Hamburg FOCUS scenario has a flux bottom boundary that is a function of the groundwater level set at −200 cm depth. In order to reduce the computational cost of simulating a 200-cm-long profile in R-SWMS, prescribed water potentials obtained from PEARL were applied as bottom BC at −145 cm depth.

Daily precipitation, potential evaporation, and transpiration fluxes were extracted from the PEARL model and used as climatic input in R-SWMS. At the soil surface, precipitation and evaporation fluxes were added in a single net top boundary flux (Fig. S4). At the root collar, a daily potential transpiration flux was imposed (Fig. S4), which switches to a constant water potential when a critical collar potential of −15000 cm is reached. The modeling period lasted from May 4th until September 20th of the first year after the warm-up.

### Soil and solute characteristics

The Hamburg scenario has a sandy loam in the top horizon and sandy soil in the lower horizons. Organic matter is 2.6 % in the top horizon and decreases with depth. Soil hydraulic properties obtained from Boesten et al. (2000) are summarized in Table S3. Four neutral organic dummy substance parameter sets are defined by FOCUS that the user can apply to test runs of the FOCUS models. Dummy solute B was chosen, as it is only marginally volatile and is readily degradable (see Table S4 for parameter set). Similarly to the PEARL model, we simulated solute degradation in the soil as a first-order process dependent on soil moisture, temperature, and soil depths (see SI for further information). Solute degradation in the root system was not modeled. Finally, based on an analysis of root retardation and the low logKow of compound B (Trapp, 2000), root sorption was not considered.

We set two main simulation scenarios to compare our model results to the PEARL model.

### Scenario 1: single root

The PEARL Hamburg scenario considers a uniform root length density with depth, with a rooting depth that increases linearly from 0 cm at the start of the growing season to 100 cm by 89 days after planting (DAP). After DAP 89, the rooting depth remains constant. To represent the root system similarly, we first performed simulations with a single vertical root and the same root growth as in PEARL. The root was placed in a 0.9 × 0.9 × 145 cm^3^ soil domain with a discretization of 0.1 × 0.1 × 1 cm^3^, representing a corresponding root length density of about 1.2 cm cm^-3^. Root hydraulic parameterization was manually tuned to obtain a uniform standard uptake fraction (SUF), which is the root water uptake distribution under uniform water potential (Couvreur et al., 2012). Root radial conductivity was set to 2 × 10^-4^ cm d^-1^ cm^-1^ and axial conductance to 5 × 10^-1^ cm^4^ d^-1^ cm^-1^.

The solute uptake properties were chosen as in PEARL, with a PUF or ε equal to 0.5 (Table S4). In addition, we performed four single root simulations: one with fully advective uptake (ε = 1) and three with diffusive uptake. We tested two small root permeabilities (P = 0.01 and 0.1 cm d^-1^) and a root permeability of 13.4 cm d^-1^ computed based on the solute’s lipophilic properties according to Eqs. (8) and (9) (see also Table S4).

### Scenario 2: complex root system architecture

Finally, simulations with a detailed 3D root system architecture were performed. In order to be comparable with the PEARL model, the root system had to have a uniform relative root length distribution with depth. The evolution of the root system depth with time was similar to the root growth rate in PEARL. The maize root system with first- and second-order laterals was modeled with the root architecture model CRootBox (Schnepf et al., 2018), with maize root growth parameters obtained from Leitner et al. (2010) (Fig. 1b).

**Fig. 2:**
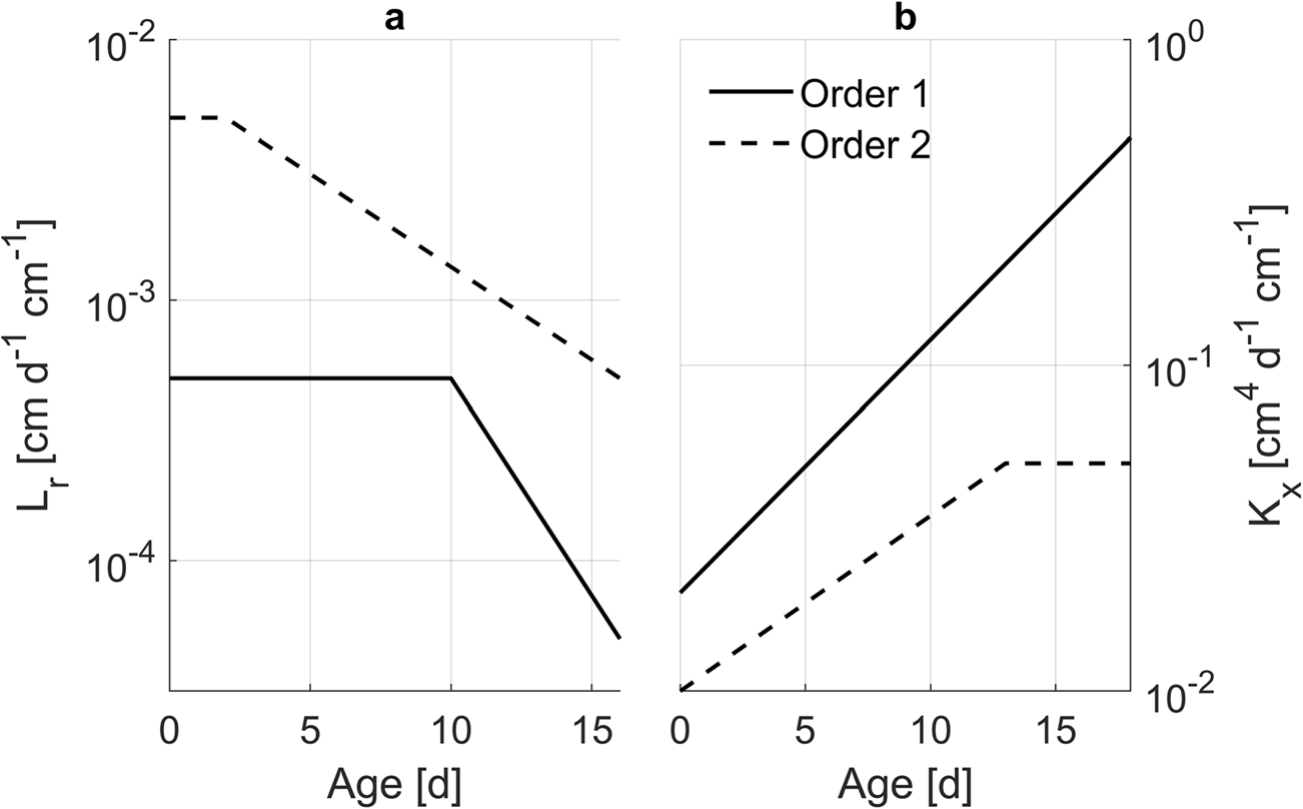
Radial conductivity, L_r_ (**a**), and axial conductance, K_x_ (**b**), as a function of age and root order for the complex root system architecture scenario

Based on typical maize plant density, the model domain was 75 × 15 × 145 cm^3^ with a discretization of 1.0 × 1.0 × 1.0 cm^3^. The resulting root length density was about 0.1 cm cm^-3^. This low root length density is explained by the lack of third order laterals, whose presence would imply larger computational times. Root hydraulic properties were parameterized as a function of age and root order based on Meunier et al. (2018) as depicted in Fig. 2.

A periodic BC was used to simulate water and solute fluxes and the root system in x and y directions, which allows us to represent field conditions.

A simulation for the whole growing season with advective uptake (ε = 0.5) was performed for comparison to single root simulations. A maximum time step (dt_max_) of 0.1 days was allowed to reduce the computational time. A comparison to a simulation run with dt_max_ = 0.01 days for the first 30 simulated days showed a good agreement and supported the appropriateness of a larger dt_max_. In addition, in order to demonstrate the effects of simulating uptake as a diffusive process, we carried out a simulation with P = 13.4 cm d^-1^, derived from the compound properties. Based on the convergence study, we expect simulations with high permeability to give similar results to simulations with fully advective uptake (ε = 1). Thus, we also run a simulation with ε = 1. The initial conditions (water potentials and solute concentrations in both root and soil) were extracted from the complex maize simulation with ε = 0.5 at 40 DAP. Simulations were run for one week and compared to the reference scenario (ε = 0.5). These simulations were run with a dt_max_ = 0.01 days as a larger dt_max_ would lead to large errors in the diffusive uptake scenario.

For all scenarios, we computed daily values of TSCF according to Eq. (3).

## Results and discussion

### Scenario 1: single root

**Fig. 3:**
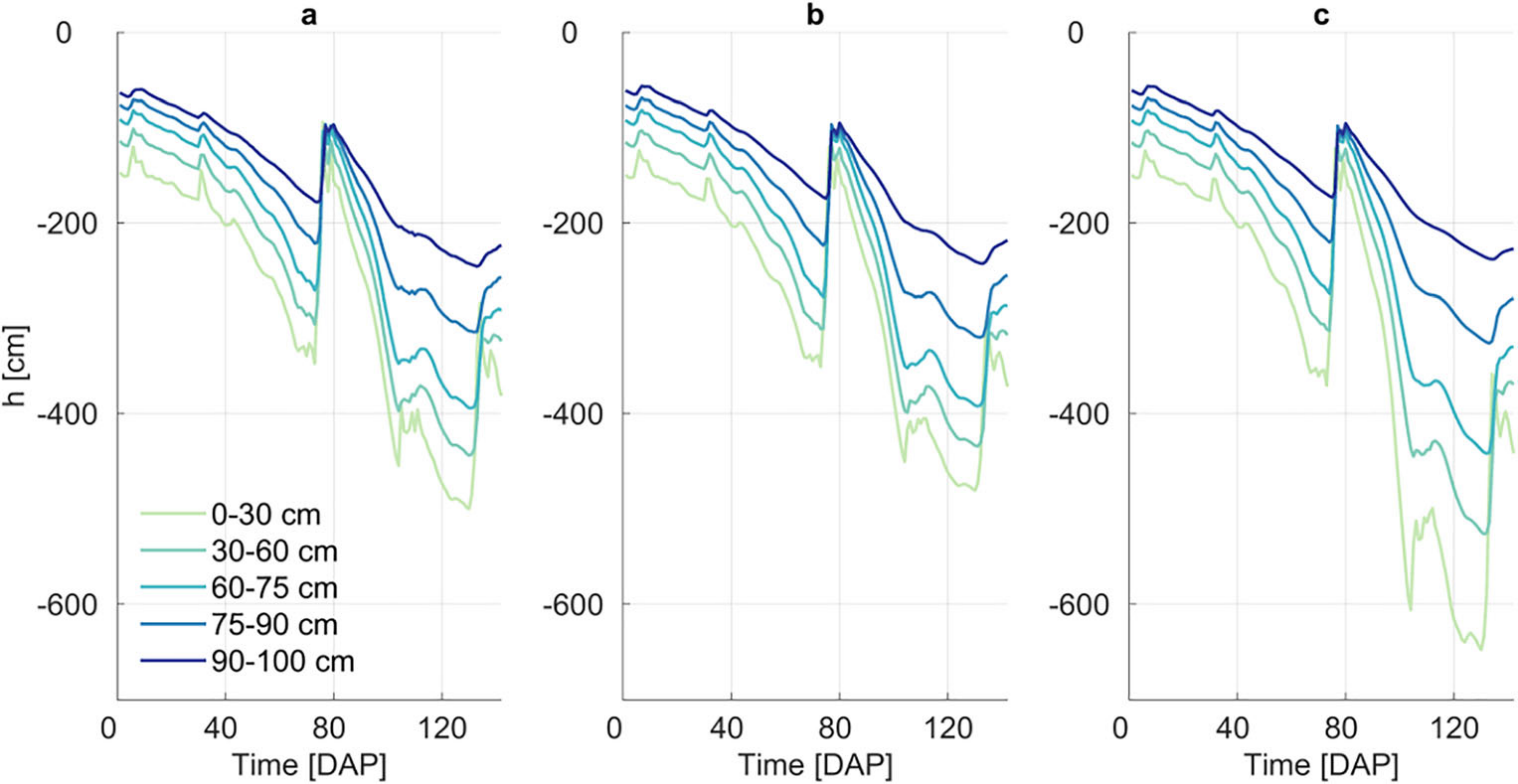
Matric water potential results for 5 soil layers (0–30 cm, 30–60 cm, 60–75 cm, 75–90 cm, and 90–100 cm) from PEARL (**a**), R-SWMS single root scenario (**b**), and R-SWMS complex root system architecture scenario (**c**) simulations

Daily values of matric potential averaged for each soil layer in the root zone are presented in Fig. 3. Matric potential decreased until about 70 DAP due to the absence of precipitation and increasing transpiration demand (see Fig. S4). Matric potential increased to almost initial values at 80 DAP due to a large rainfall event, and went on to decrease again during a prolonged dry period. These data are well in agreement with results obtained by PEARL.

**Fig. 4:**
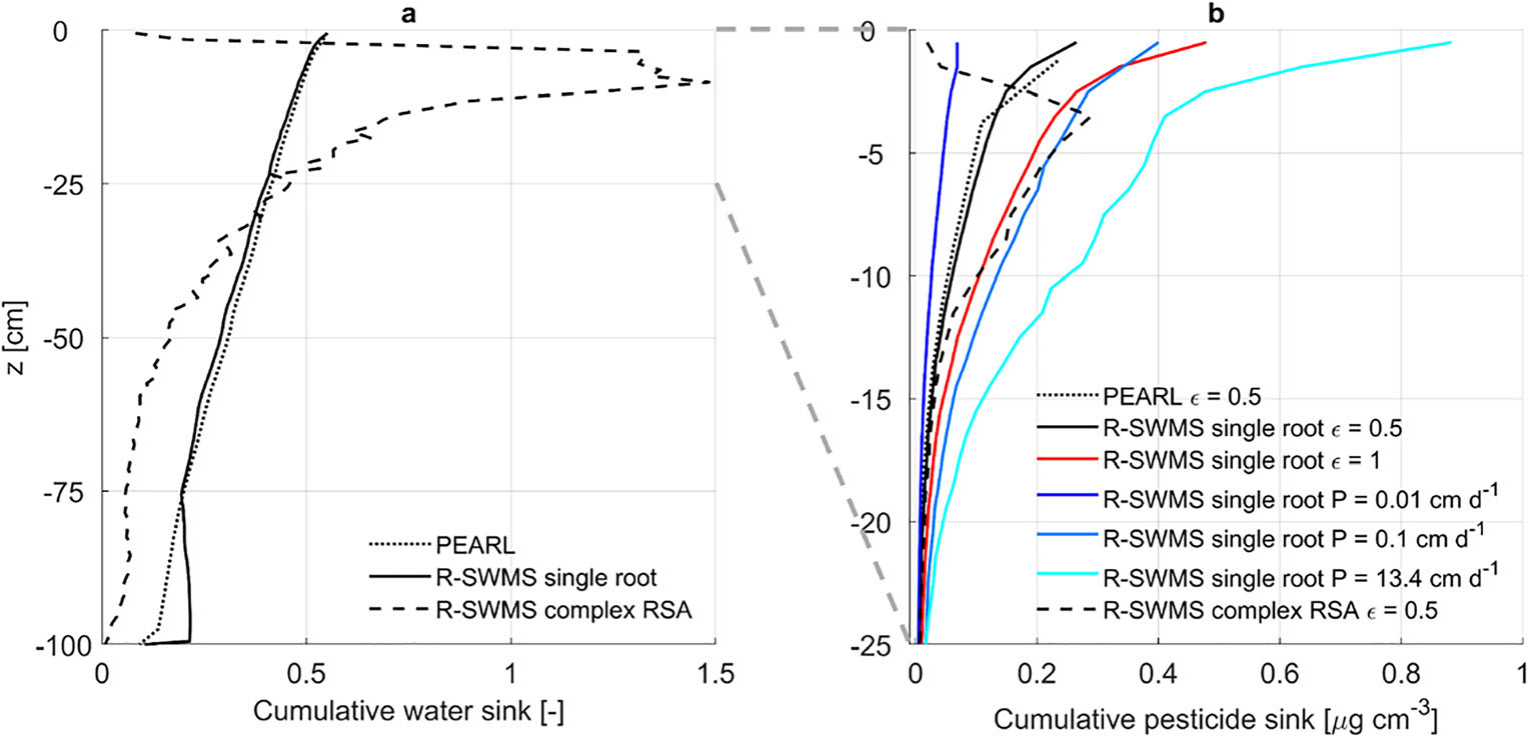
Cumulative water (**a**) and pesticide (**b**) sink distribution for PEARL (dotted line), the single root R-SWMS (solid line), and complex root system architecture (RSA) R-SWMS (dashed line) scenarios. Root solute uptake is modeled as an advective mechanism with ε = 0.5 (black), ε = 1 (red) and as a diffusive mechanism with P = 0.01 (dark blue), P = 0.1 (blue) and P = 13.4 cm d^-1^ (cyan)

The cumulative sink distribution (Fig. 4a) shows similar water uptake patterns between PEARL and R-SWMS single root simulation. A slightly larger uptake at the bottom was simulated with R-SWMS during dry periods. Whereas PEARL cannot simulate compensatory root water uptake, R-SWMS reduces water uptake in zones with lower water potentials while increasing it in wetter areas. This compensation process arises from the water flow equations in R-SWMS, which calculate the water uptake rate based on water potential gradients between soil and root.

**Fig. 5:**
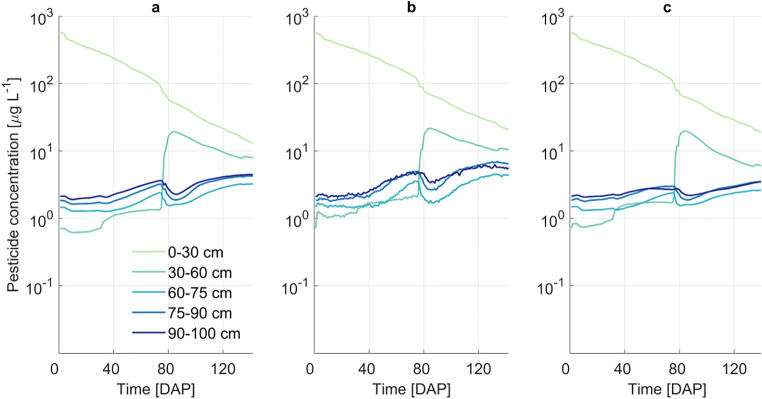
Dissolved soil pesticide concentration results for 5 soil layers (0–30 cm, 30–60 cm, 60–75 cm, 75–90 cm, and 90–100 cm) from PEARL (**a**), R-SWMS single root (**b**), and R-SWMS complex root system architecture (**c**) scenario simulations. Solute uptake is modeled as an advective mechanism with ε = 0.5

Soil pesticide concentrations showed similar trends for both models (Fig. 5a, b). Mean concentrations in the first soil layer (0–30 cm) decreased with time due to root water uptake, degradation, and leaching to deeper layers. Concentration in the second soil layer (30–60 cm) substantially increased after the large rainfall event 80 DAP due to leaching from the upper most layer. In the bottom layers, pesticide concentration increased during dry periods due to root water uptake and upward transport of pesticide from below the root zone.

In both R-SWMS and PEARL simulations, most pesticide uptake took place in the upper 25 cm of the soil (Fig. 4b) as a result of root water uptake and pesticide concentration distributions. About 0.17 kg ha^-1^ of pesticide reached the root collar with R-SWMS when simulating uptake as an advective process with ε = 0.5 (Fig. 4a). This is close to the 0.16 kg ha^-1^ of pesticide uptake simulated with PEARL. The close agreement of our single root simulation with ε = 0.5 to the results from PEARL demonstrates that the implementation of soil processes in our model was done correctly. When increasing ε to 1.0, the total pesticide uptake nearly doubled (0.3 kg ha^-1^).

**Fig. 6:**
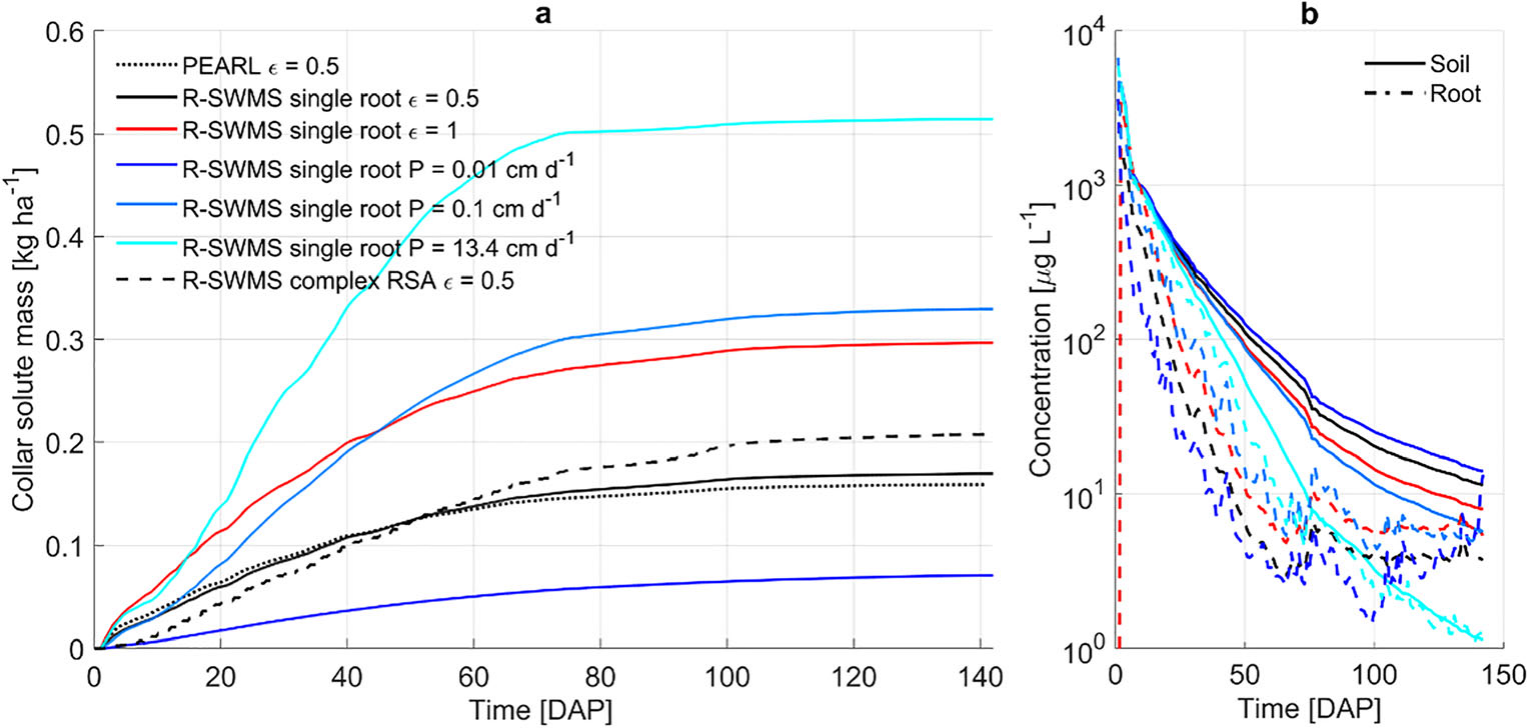
**a** Simulated collar solute mass with PEARL (dotted line), the single root R-SWMS (solid line), and complex root system architecture (RSA) R-SWMS (dashed line) scenarios. Root solute uptake is modeled as an advective mechanism with ε = 0.5 (black), ε = 1 (red), and as a diffusive mechanism with P = 0.01 cm d^-1^ (dark blue), P = 0.1 cm d^-1^ (blue), and P = 13.4 cm d^-1^ (cyan). **b** Soil (solid lines) and root (dashed lines) average concentrations for R-SWMS single root simulations under different solute uptake mechanisms. Soil and solute concentrations are computed for soil elements and root segments, respectively, from the soil surface down to the maximum rooting depth of each day

When simulating pesticide uptake as a diffusive mechanism with P = 13.4 cm d^-1^, computed according to root membrane and solute properties, pesticide uptake was about 0.51 kg ha^-1^, larger than when simulating uptake as an advective mechanism with ε = 1. Until about 15 DAP, pesticide uptake was similar for both scenarios. However, as the root system grew, it took up water from deeper soil layers with lower pesticide concentration, which had a dilution effect on the root water concentrations in the upper part of the root system. As a consequence, solute got flushed from the root system into the collar, stimulating further uptake of solute from the upper root zone in the diffusive uptake scenario, whereas in the advective uptake scenario, as solute uptake increased in lower root zones due to the shift in water uptake, the ratio of solute uptake in the upper root zone decreased. This is further illustrated when comparing average root and soil concentrations (Fig. 6b). While root and soil concentrations are very close for the diffusive uptake scenario, concentrations in the root are lower than in the soil for the advective uptake scenario.

When simulating diffusive uptake with P = 0.01 cm d^-1^, solute uptake was limited by P and the total pesticide uptake was the lowest (0.07 kg ha^-1^). With an intermediate P = 0.1 cm d^-1^, solute uptake was about 0.33 kg ha^-1^, similar to the fully advective uptake scenario (Fig. 6a).

### Scenario 2: complex root system architecture

**Fig. 7:**
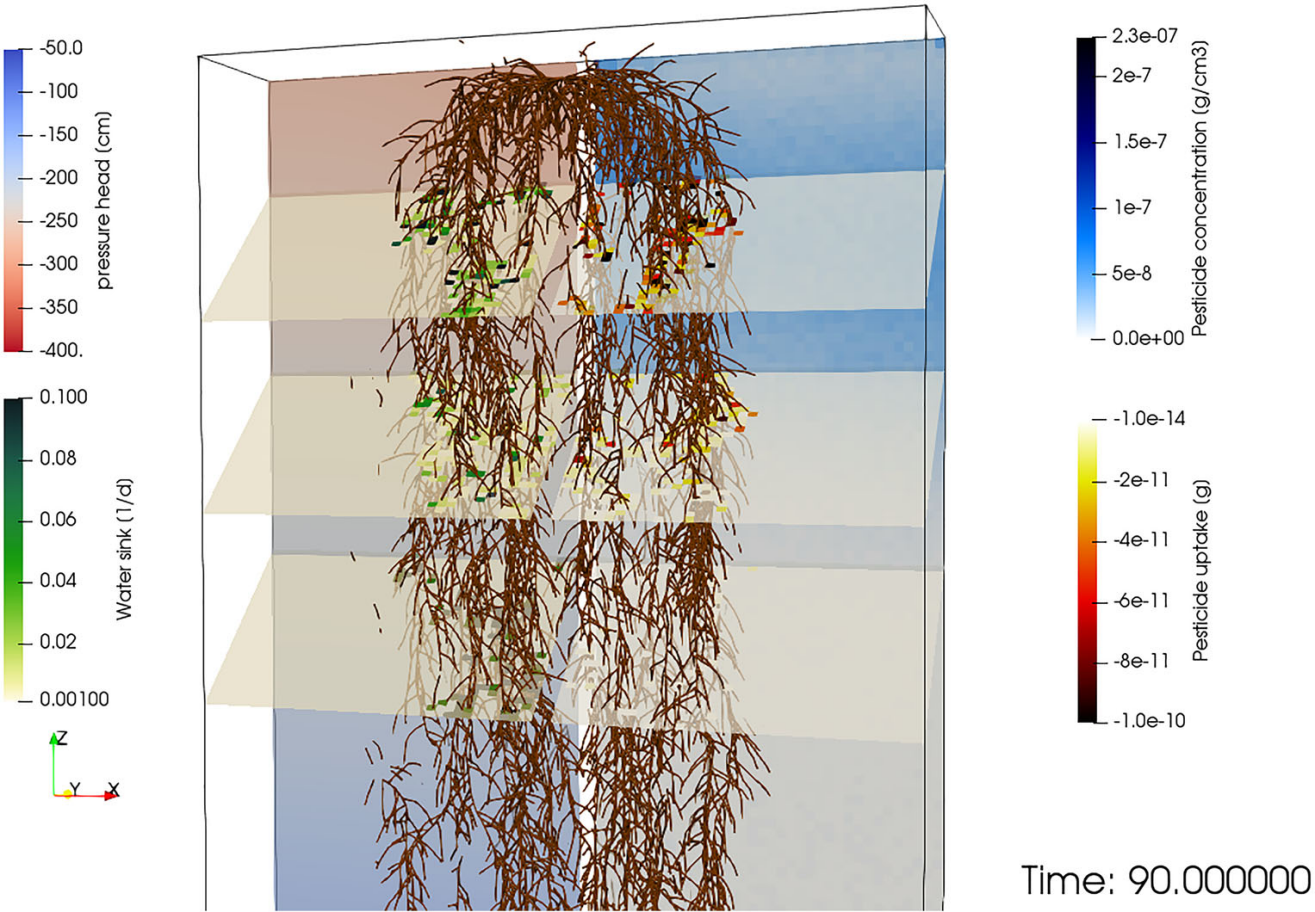
2D projection of the 3D distribution of matric potential [cm] (left panel) and pesticide concentration [g cm^-3^] in the soil (right panel) 90 days after planting (DAP), together with the 2D distribution of water [d^-1^] (left panel) and pesticide [g cm^-3^] (right panel) sinks at − 20, − 40, and − 60 cm depth for a simulation performed with a complex root system architecture. Root solute uptake was modeled as an advective mechanism with ε = 0.5

Figure 7 presents a snapshot of the maize simulation 90 DAP, showing root water uptake mostly taking place at the top and fringes of the root system, whereas pesticide was mostly taken up at the top of the root system. A video of the simulation is available in the SI.

Cumulative water uptake simulated with the complex root system architecture in the top 25 cm was 1.75 times the water uptake simulated with a single root (Fig. 4a), resulting in 1.25 times larger solute uptake from the top soil (Figs. 4b and 6). Although root length was quite uniform with depth, the root hydraulic parameterization resulted in a maize plant that predominantly took up water from the top soil, as shown with the SUF in Fig. 1c.

From 80 to 130 DAP, root water uptake in the top 25 cm was almost 3 times larger than when simulating a single root, which led to a larger drop in soil water potential when compared to PEARL and the single root R-SWMS simulation (Fig. 3). This triggered upward water flow, causing the soil water potential in the second and third layers to drop further as well.

As in the single root simulations, the 1-week simulations showed larger solute uptake when simulating uptake as a diffusive process (Fig. S5). Root water uptake from deeper soil layers, with lower pesticide concentration, flushed out the solute from the upper part of the root, leading to larger solute uptake when simulating diffusive uptake.

**Fig. 8:**
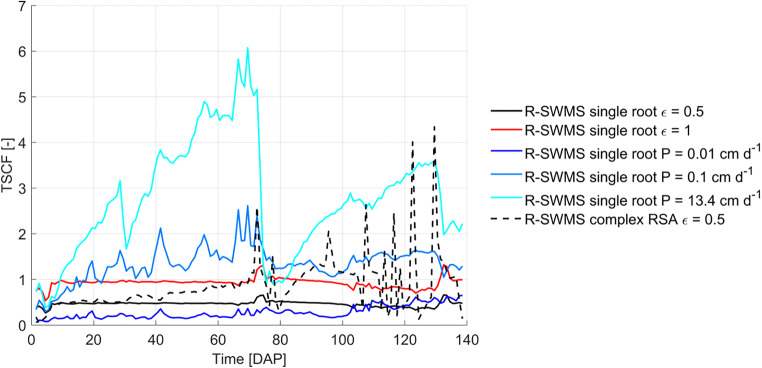
Daily values of transpiration stream concentration factor (TSCF). TSCF is calculated as collar concentrations divided by mean soil concentration. Mean soil concentration was calculated for elements down to the depth corresponding to the maximum rooting depth at each day

Finally, when uptake was simulated as an advective process, daily TSCF values remained close to ε (Fig. 8). Deviation from ε occured when soil average concentrations differed from flux-averaged concentrations, showing the latter is more appropriate for TSCF calculations. Moreover, TSCF values above 1 were obtained when diffusive uptake was considered as a result of the diluting effect described above. Most studies of plant uptake and bioaccumulation of organic substances report experimental TSCF values that range from 0 to 1 (Doucette et al., 2017), and values above 1 have been explained by active uptake mechanisms (Briggs et al., 1987). However, 80% of these studies are performed in hydroponic setups where dilution effects cannot occur due to the uniform distribution of substances.

## Conclusions

From simulations that were carried out to test the model implementation (see Figs. S2 and S3), we observed that when root water uptake takes place in soil with homogenous solute concentration, diffusive uptake can be described as an advective uptake process if the diffusion rates are sufficiently high so that an equilibrium between root and soil concentrations can be assumed. However, under heterogeneous soil solute concentrations, the distribution of water uptake with depth and the mechanism chosen to describe solute uptake will play an important role in determining the total plant uptake. In our tested scenario, a sharp concentration gradient in the soil is simulated, with most solute located at the top of the profile. Due to the high permeability of the membrane to compound B, a fast equilibrium is established between root and soil concentrations when diffusive uptake is considered. As the root system takes up water from the bottom of the profile, with low solute concentration, and water gets transported to the collar, solute concentration in the root system decrease, thereby enhancing the transport of solute from the soil to the root. This phenomenon would not occur under an inverted solute concentration gradient or under a uniform solute distribution; in such cases, approximating diffusive uptake with an advective mechanism will be sufficient to estimate solute uptake.

Although the transfer of solutes towards newly grown segments at the root tips when these segments emerge was not considered, this process is likely to be only marginally relevant since solute was directly applied to the soil. However, such transport would be of relevance when plant roots contain initially the substance, such as in pesticide application through seed treatment. Nevertheless, advective transport towards root tips is included in the model and may occur when water flows downwards in the root system. This may for instance occur after rainfall events in summer that wet up the top soil layer but not the dried out deeper soil layers.

The newly implemented model allows one to simulate solute uptake by plants in a more mechanistic way. The impact of the different uptake mechanisms on pesticide fate can be represented in the model simulations and related to properties of the plant and the substance. The model also enables a better representation of root hydraulics and their link to root water uptake, which can affect water uptake profiles in the soil and, subsequently, pesticide uptake. However, its limitation to neutral compounds, the large computational demand when simulating large root architectural systems, and the laborious parameterization of root hydraulics remain a challenge.

Although its application in this work was limited to pesticide uptake, this model can simulate the fate of other organic pollutants and can be used to investigate the capability of plants with different root system architectures and hydraulic properties for the phytoremediation of contaminated soils.

## Supplementary Information

ESM 1 (PDF 875 kb)

ESM 2

## Data Availability

The datasets used and/or analyzed during the current study are available from the corresponding author on reasonable request.
