# Supplementary material for: Mechanistic modeling of pesticide uptake with a 3D plant architecture model
Source: Environ Sci Pollut Res Int. 2021 Jun 17;28(39):55678–89. doi: 10.1007/s11356-021-14878-3 (PMC8494688; doi:10.1007/s11356-021-14878-3)
Supplement: Supplementary file 1 — (PDF 875 kb) [file 11356_2021_14878_MOESM1_ESM.pdf]

Supplementary Information

Manuscript title: Mechanistic modeling of pesticide uptake with a 3D plant architecture model

Journal name: Environmental Science and Pollution Research

Author names: Helena Jorda*; Katrin Huber; Asta Kunkel; Jan Vanderborght; Mathieu Javaux; Christoph Oberdörster; Klaus Hammel; Andrea Schnepf.

*Corresponding author. Present address: Institute of Bio- and Geosciences, Agrosphere Institute, IBG-3, Forschungszentrum Jülich GmbH, 52428 Jülich, Germany. Email address: h.jorda@fz-juelich.de.

A. Compound degradation theory

The reference degradation rate constant $k_{S,r}$ [T^{-1}] is determined from

$$k_{S,r} = \ln(2)/DT50 \qquad \text{(S1)}$$

where DT50 [T] is the half-life time of the compound. The actual degradation rate constant is then determined by correcting for the effects of temperature f_t [-], soil moisture f_m [-], and soil depth f_d [-]:

$$k_S = f_t f_m f_d k_{S,r}. \qquad \text{(S2)}$$

The temperature correction f_t is described by

$$f_t = e^{\frac{-E_a}{R_g}\left(\frac{1}{T}-\frac{1}{T_r}\right)}, \qquad \text{(S3)}$$

where E_a [J mol-1] is the molar activation energy, R_g [J mol^{-1} K^{-1}] the molar gas constant, T the actual temperature [K] and T_r the reference temperature [K] taken to be 293 K.

The dependency of degradation on moisture is described by

$$f_m = min\left(1, \left(\frac{\theta_S}{\theta_{fc}}\right)^B\right), \qquad \text{(S4)}$$

where B [-] is an empirical factor, θ_s [-] is the soil water content, and θ_{fc} is the soil moisture at field capacity, which is defined as θ_s at 100 hPa. B is set to 0.7 according to Boesten et al. (2000).

The factor f_d is a given in Table S2 (Boesten et al., 2000), but may vary in the scenarios due to variations in soil layer depth.

B. Numerical implementation

B1. Operator splitting method for coupling R-SWMS with ParTrace

To solve pesticide transport in the soil and the root, we updated the coupled version of R-SWMS for water flow in soil and root, and for solute transport within the root (Huber et al., 2014) on the one hand, with ParTrace for solute transport in the soil domain on the other hand. A coupled version of R-SWMS with ParTrace was firstly developed by Schröder (Schröder et al., 2013, 2012), but this version only considered advective uptake or active uptake so that concentrations of the substance in the root did not influence the uptake calculations and root concentrations were not calculated. In the current version, solute transport in the soil and in the root system were coupled. The two models were coupled using a *non-iterative sequential approach* (Jacques et al., 2006), where the single operators are ordered and solved subsequently, and the solution is updated in that order.

The right hand sides of the transport equations in soil and root (Eq. 2 and 5 in the main text) consist of three terms or 'operators' that each define a change of the solute mass per unit of time in the soil or root due to a specific process or 'operation'. The first term represents the changes due to transport (transport operator: Tran), the second due to degradation processes (degradation operator: Deg) and the third due to exchanges between the soil and root that act a source or sink terms (uptake operator: S). Although these processes act simultaneously, in the

sequential split method, these operators are carried out sequentially to calculate the change of the solute concentration during a time step.

In the soil, solute concentration is first updated according to the degradation operator, Deg_S. The resulting concentration is updated according to solute uptake operator, S_S; and finally, solute concentration is modified according to the transport operator, $Tran_S$. Equations S5-S7 describe how the solution numerically advances from one time step (*j*) to the next (*j+1*):

$$C_{s,Deg}^{j+1} = C_s^j + \Delta t\, Deg_S(C_s^j) \tag{S5}$$

$$C_{s,Deg,S}^{j+1} = C_{s,Deg}^{j+1} + \Delta t\, S_S(C_{s,Deg}^{j+1}, C_{R,Deg}^{j+1}) \tag{S6}$$

$$C_{s,Deg,S,Tran}^{j+1} = C_{s,Deg,S}^{j+1} + \Delta t\, Tran_S(C_{s,Deg,S}^{j+1}) \tag{S7}$$

where Δt is the coupling time step, $C_{s,Deg}^{j+1}$ is the concentration in the soil after calculating degradation, C_S^j is the concentration in the soil at the beginning of time step j, $C_{s,Deg,S}^{j+1}$ is the concentration in the soil after calculating degradation and uptake, $C_{R,Deg}^{j+1}$ is the concentration in the root after degradation is calculated in the root, and $C_{s,Deg,S,Tran}^{j+1}$ is the concentration in the soil after degradation, uptake and transport are calculated. Water contents and fluxes, which determine degradation, exchange between soil and root, and transport, at the beginning of the time step are used.

Regarding the root, the same order of operators is followed. The corresponding equations of the operator scheme for the root system is

$$C_{R,Deg}^{j+1} = C_R^j + \Delta t\, Deg_R(C_R^j) \tag{S8}$$

$$C_{R,Deg,S}^{j+1} = C_{R,Deg}^{j+1} + \Delta t\, S_R(C_{R,Deg}^{j+1}, C_{S,Deg}^{j+1}) \tag{S9}$$

$$C_{R,Deg,S,Tran}^{j+1} = C_{R,Deg,S}^{j+1} + \Delta t\, Tran_R(C_{R,Deg,S}^{j+1}) \tag{S10}$$

where $C_{R,Deg}^{j+1}$ is the concentration in the root after calculating degradation, C_R^j is the concentration in the root at the beginning of time step j, $C_{R,Deg,S}^{j+1}$ is the concentration in the root

after calculating degradation and uptake, and $C_{R,Deg,S,Tran}^{j+1}$ is the concentration in the soil after degradation, uptake and transport are calculated.

A particle tracking method is used to carry out the transport operators in the soil and in the root. For the soil domain, we refer to Schröder et al. (Schröder et al., 2013, 2012) and Bechtold et al. (2011) for more detailed information about the implementation of the particle tracker. In the following we discuss the implementations of the exchange between the soil and root domains, the transport within the root domain and the particular case of non-linear sorption.

B2. Solute uptake by roots

Written in terms of mass, the solute uptake of one root segment *i* in any given soil voxel during a defined time step Δt is obtained by

$$m_{u,i} = \left(C_{S,l} - C_{R,l,i}\right) A_{R,i} P_i \Delta t + \varepsilon_i J_{w,r,i} A_{R,i} C_{S,l} \Delta t \qquad \text{for} \qquad J_{w,r,i} > 0, \quad \text{(S11)}$$

$$m_{u,i} = \left(C_{S,l} - C_{R,l,i}\right) A_{R,i} P_i \Delta t + \varepsilon_i J_{w,r,i} A_{R,i} C_{R,l,i} \Delta t \qquad \text{for} \qquad J_{w,r,i} < 0, \quad \text{(S12)}$$

where $m_{u,i}$ [M] is the root solute uptake by segment *i* during time step Δt [T].

When P_i is relatively large, a very small Δt is necessary to prevent oscillations in solute mass in the root. In order to avoid having to use very small Δt, an equilibrium approach was implemented to limit the amount of mass that could be transferred by diffusive uptake during a defined time step Δt. The maximum mass of solutes than can be taken up by diffusive uptake was defined based on the value at which an equilibrium between soil and root solute concentration is reached.

First, we defined the mass of solute in the soil and root volumes:

$$m_S = \left(\theta_S C_{S,l} + K_{D,S} \rho_{b,S} C_{S,l}\right) V_S, \qquad \text{(S13)}$$

$$m_{R,i} = \left(\theta_{R,i} C_{R,l,i} + K_{D,R,i} \rho_{b,R,i} C_{R,l,i}\right) V_{R,i}, \qquad \text{(S14)}$$

where m_S [M] is the total mass in the surrounding soil voxel and $m_{R,i}$ [M] is the total solute mass in root segment *i*.

By the end of a time step Δt, the maximum uptake mass by diffusive uptake was defined based on reaching an equilibrium between soil and root solute concentration, while maintaining mass balance in the system. At equilibrium, the concentrations in root and soil are equal, i.e.,

$$C_{S,l} = C_{R,l,i} = C_{eq}, \text{ and} \tag{S15}$$

$$m_S + \sum_{i=1}^{n} m_{R,i} = m_{S,0} + \sum_{i=1}^{n} m_{R,i,0}, \tag{S16}$$

where $m_{S,0}$ and $m_{R,i,0}$ are the masses in the soil and the root at the beginning of the time step, respectively, and n is the number of root segments inside the given soil voxel.

The previous two equations can be combined into

$$C_{eq}\left[\left(\theta_S + K_{D,S}\rho_{b,S}\right)V_S + \sum_{i=1}^{n}\left(\theta_{R,i} + K_{D,R,i}\rho_{b,R,i}\right)V_{R,i}\right] = m_{S,0} + \sum_{i=1}^{n} m_{R,i,0}, \tag{S17}$$

where C_{eq} is the root and soil concentrations at equilibrium.

Given the above equations, we can calculate the maximum uptake mass by diffusive uptake in a given soil element, $m_{u,dif,max}$ [M] as

$$m_{u,dif,max} = m_{S,0} - m_S = m_{S,0} - C_{eq}\left(\theta_S + K_{D,S}\rho_{b,S}\right)V_S, \tag{S18}$$

$$m_{u,dif,max} = m_{S,0} - \left(\theta_S + K_{D,S}\rho_{b,S}\right)V_S \frac{m_{S,0} + \sum_{i=1}^{n} m_{R,i,0}}{\left[\left(\theta_S + K_{D,S}\rho_{b,S}\right)V_S + \sum_{i=1}^{n}\left(\theta_{R,i} + K_{D,R,i}\right)V_{R,i}\right]}. \tag{S19}$$

And the maximum uptake mass by diffusive uptake per root segment, $m_{u,dif,max,i}$, as

$$m_{u,dif,max,i} = \left(\theta_{R,i} + K_{D,R,i}\rho_{b,R,i}\right)V_{R,i} \frac{m_{S,0} + \sum_{i=1}^{n} m_{R,i,0}}{\left[\left(\theta_S + K_{D,S}\rho_{b,S}\right)V_S + \sum_{i=1}^{n}\left(\theta_{R,i} + K_{D,R,i}\right)V_{R,i}\right]} - m_{R,i,0}. \tag{S20}$$

When Freundlich sorption is assumed in soils and/or roots, the equilibrium concentration needs to be found numerically, i.e., via the Newton method, where

$$f\left(C_{eq}\right) = m_{S,0} + \sum_{i=1}^{n} m_{R,i,0} - \left(\theta_S C_{eq} + K_{F,S}\rho_{b,S}{C_{eq}}^{n_S}\right)V_S - \sum_{i=1}^{n}\left(\theta_{R,i} C_{eq} + K_{F,R,i}\rho_{b,R,i}{C_{eq}}^{n_R}\right)V_{R,i}, \tag{S21}$$

and

$$f'\left(C_{eq}\right) = -\left(\theta_S + n_S K_{F,S} \rho_{b,S} {C_{eq}}^{n_S - 1}\right) V_S - \sum_{i=1}^{n} \left(\theta_{R,i} + n_R K_{F,R,i} \rho_{b,R,i} {C_{eq}}^{n_R - 1}\right) V_{R,i}, \qquad \text{(S22)}$$

where $K_{F,S}$ [L^3 M^{-1} (M L^{-3})$^{1-n}$] and $K_{F,R}$ [L^3 M^{-1} (M L^{-3})$^{1-n}$] are the Freundlich coefficients in soil and root, respectively, and n_S [-] and n_R [-] are the Freundlich exponents in soil and root, respectively.

The next iterative solution can be found

$$C_{eq,k+1} = C_{eq,k} - \frac{f\left(C_{eq,k}\right)}{f'\left(C_{eq,k}\right)}, \qquad \text{(S23)}$$

where k is the iteration number.

The iterative process is stopped when the convergence criterion $|f/C_{eq}| \leq 1e\text{-}5$ is reached, with

$$f = \left|\frac{C_{eq,k} - C_{eq,k+1}}{C_{eq,k}}\right|. \qquad \text{(S24)}$$

Once m_u is calculated for each root segment and for a determined time step, a constant amount of particles is created and inserted in each root segment. The amount of inserted particles is determined before the start of the simulation. Therefore, the mass associated to each particle is defined by m_u.

If the mass is released from the root to the soil (m_u<0), the particles' mass within the root segment is decreased according to an R_{fact} [-] obtained by

$$R_{fact} = 1 - \frac{\left|m_{u,i}\right|}{m_i}, \qquad \text{(S25)}$$

where m_i [M] is the total solute mass in the respective segment and $m_{u,i}$ [M] the mass leaving the root segment.

Similarly, the mass of the particles in the soil is increased or decreased by multiplying the mass with a S_{fact} [-] obtained by

$$S_{fact} = 1 - \frac{\sum_{i=1}^{n} m_{u,i}}{m_S}. \qquad \text{(S26)}$$

B3. Solute transport within the roots

Once entered the root, the solute particles are transported with the advective water flow within the roots. Particles are moved along a root segment by a distance

$$\mathrm{d_{p,i}} = \frac{\mathrm{v}_{R,\mathrm{p,i}}\Delta\mathrm{t}}{\mathrm{R}_{R,i}}, \tag{S27}$$

where $d_{p,i}$ [L] is the particle displacement in root segment i, $v_{R,p,i}$ is the velocity [L T^{-1}] in the root segment i in which particle p is located, and $R_{R,i}$ is the root retardation factor in root segment i [-].

In the case that $\mathrm{d_{p,i}}$ is larger than the remaining length within the root segment i, the particle moves into the next root segment. The remaining travel distance for the particle is calculated based on the remaining travel time, the velocity, and the retardation factor of the next segment. When a particle reaches the root collar, its mass is stored in a cumulative variable and the particle is removed from the system.

In case of reverse water flow, e.g. during night, particles may flow backwards. When reaching a root tip, the particles cannot exit into the soil as the water velocity at the root tips is zero.

B4. Non-linear sorption in soil and roots

Solutes occur either in the sorbed or the dissolved phase. Only the dissolved solutes can move freely within the medium. For a particle tracking algorithm there are two ways to describe the impact of sorption on transport. Either a single particle can be in its sorbed or dissolved state, which would, in case of high sorption, lead to a substantial reduction of the amount of particles moving in the system. The second option is that all particles move at a slower velocity and diffuse slower in the soil, which will conserve the number of particles. Due to this reason, we chose to implement the latter option.

The retardation is equal to the fraction of solutes in the dissolved state compared to the total amount of solute mass in the soil/root volume. Assuming that the sorption follows the

Freundlich isotherm, the dissolved concentration in the root needs to be calculated iteratively by

$$C_{R,l,k+1} = C_{R,l,k} - \frac{C_{R,l,k} + \frac{\rho_R K_{F,R}}{\theta_R} C_{R,l,k}{}^{n} - \frac{C_{R,T,k}}{\theta_R}}{1 + n\frac{\rho_R K_{F,R}}{\theta_R} C_{R,l,k}^{(n-1)}}, \quad \text{(S28)}$$

where $C_{R,T}$ [M L^{-3}] is the total concentration in the root. The iteration is stopped when the second term on the right hand side reaches a sufficiently small value. In R-SWMS this is solved using the Newton method with the convergence criterion $|f/C_{R,l}| \leq 1e\text{-}5$,

$$f = \left|\frac{C_{R,l,k} - C_{R,l,k+1}}{C_{R,l,k}}\right|. \quad \text{(S29)}$$

B5. Convergence study

A convergence study was performed to evaluate the implementation of the new solute uptake model and the effect of space and time discretization. A finer mesh usually results in a more accurate solution, but at the cost of increasing computation time. Thus, it is of interest to find a balance such that the solution is accurate enough at a reasonable computational cost. The simulation setup consisted of a 100 cm long vertical root placed in a 0.9 x 0.9 x 100 cm^3 soil domain. This domain was based on the setup used to compare the new solute uptake model with PEARL.

Homogeneous water potential and solute concentration were set as initial conditions. Constant transpiration flux at the root collar and irrigation flux at the soil surface were imposed as top boundary conditions. The irrigation water had a constant solute concentration of 2×10^{-7} g cm^{-3}. Different values of P and ε ranging from low to high were evaluated (Table S1). Scenarios 1 to 3 were run with the equilibrium approach.

Table S1. Overall permeability (P) and ε values used in each scenario of the convergence study.

Scenario	P [cm d^{-1}]	ε [-]
1	0.01	0
2	1	0
3	100	0
4	0	0.05
5	0	0.5
6	0	1

A commonly used spatial discretization of the soil domain is in the order of 1 cm, a commonly acceptable time step is in the order of 0.1 days. Thus, we first ran each scenario for the coarsest possible discretization of Δt=0.1 and Δx=1cm; and then refined both space and time steps. Altogether, we ran 9 different time and space discretization combinations, with fixed time steps of 0.001, 0.01 and 0.1 days; and 0.1, 0.3 and 0.9 cm as horizontal spatial discretization (x and y axes). A constant discretization of 1 cm was used in the vertical axis. Simulation time was set to 5 days.

Finally, the convergence to equilibrium for solute diffusive uptake was studied (adapted from scenario 3 in Table S1). The equilibrium approach with a larger time step (0.01 days) was compared to the normal exchange approach that required smaller time steps (0.0001 days). Both simulations were then compared to a fully advective scenario. All three scenarios were run with a horizontal spatial discretization of 0.3 cm, a domain length of 6 cm and a root length of 4 cm to optimize computational resources.

The results from the convergence study indicate that time discretization plays a larger role than space discretization in terms of solute uptake accuracy. Differences in cumulative mass at the root collar were relatively small for all simulations except for those with a time step of 0.1 days (Figure S1). These results indicate that a maximum time step of 0.01 days is required to obtain accurate results in both single root (dx = 0.1 cm) and complex root architecture (dx = 1 cm ≈ 0.9 cm) simulations.

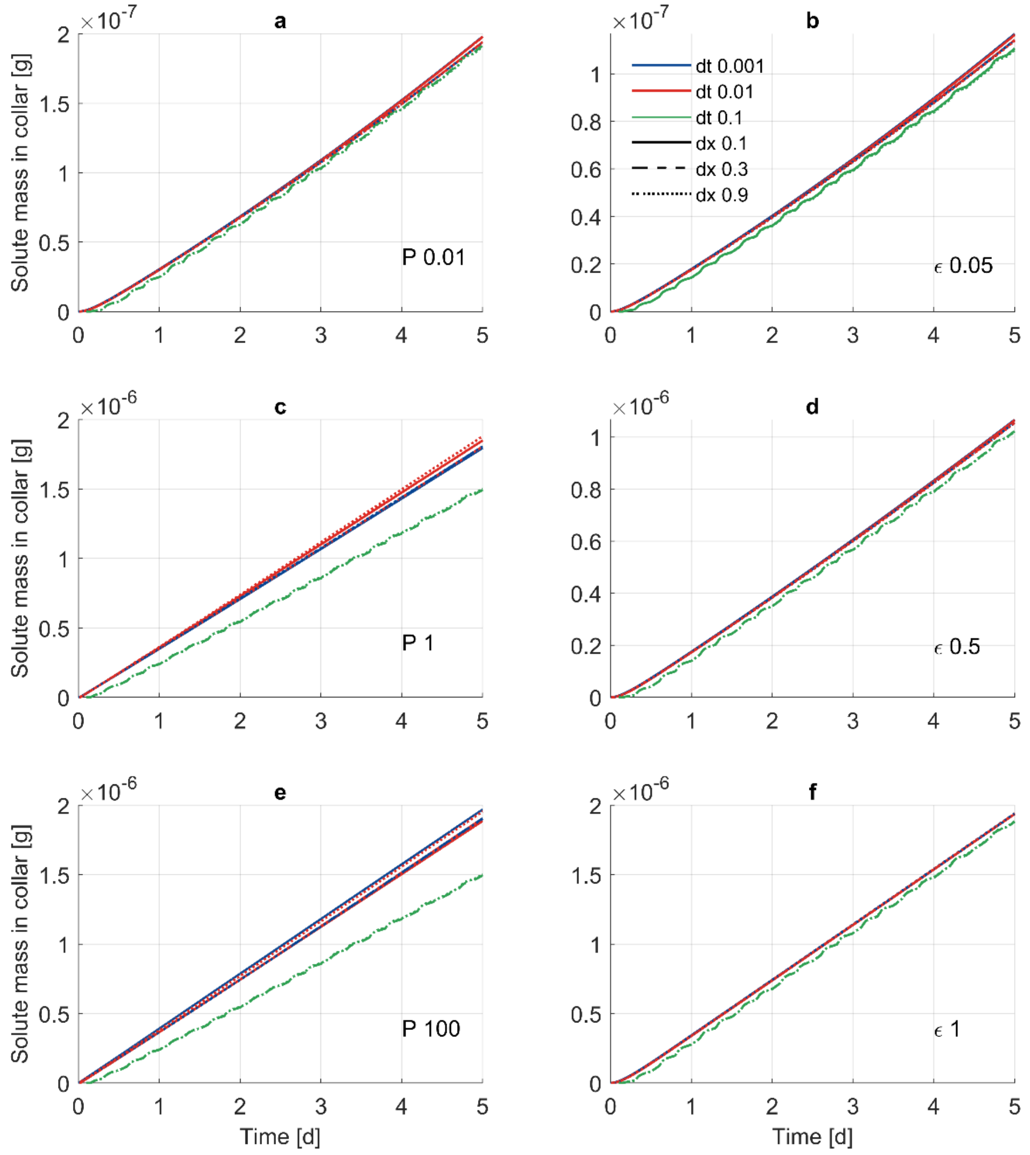

Figure S1. Cumulative solute mass that reaches the collar for scenarios 1 to 6. Plots a, c and e correspond to scenarios 1, 2 and 3, respectively; plots b, d and f correspond to scenarios 4, 5 and 6, respectively. In each scenario, we compare different time steps (blue: 0.001 d; red: 0.01 d; green: 0.1 d) and space discretization (solid: 0.1 cm; dashed: 0.3 cm; dotted: 0.9 cm).

Under fully advective uptake conditions ($\varepsilon = 1$), all dissolved solute enters the root with the water uptake flow. Given a transpiration rate of 2 cm^3 d^{-1} and an average soil concentration of 2 10^{-7} g cm^{-3}, after 5 days we should expect a cumulative solute uptake of 2 10^{-6} g, which corresponds with the sum of solute mass in the root collar and root (data not shown). In addition, root concentration is slightly lower than soil concentration (Figure S2a). This is due to the

removal of part of the solute reaching the collar at the end of the time step, while water content in the root is assumed to remain constant.

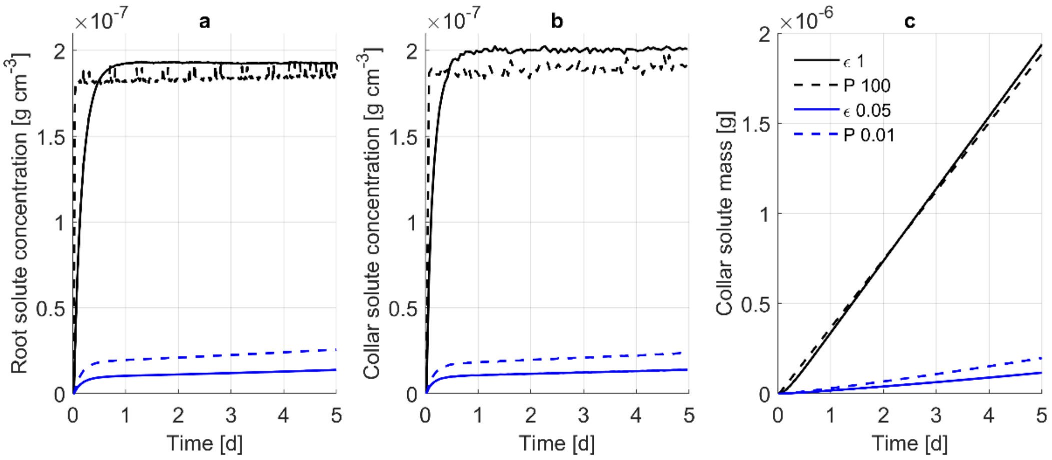

Figure S2. Comparison between simulations with partial advective uptake (ε = 0.05), fully advective uptake (ε = 1), diffusive uptake with low P (P =0.01 cm d^{-1}) and diffusive uptake with large P (P = 100 cm d^{-1}). Collar solute concentration (b) is calculated by dividing the solute mass that reaches the collar by the amount of transpired water at each time step. The results correspond to simulations with dx = 0.1 cm and dt = 0.01 days.

Simulations with large diffusive uptake (P = 100 cm d^{-1}) behave similarly to those with fully advective uptake. As solute travels from the root to the collar, concentration in the root decreases and solute uptake from soil to root takes place in order to maintain the same solute concentration in both root and soil. Figure S2a shows that simulations with fully advective uptake (ε = 1) and large diffusive uptake (P = 100 cm d^{-1}) tend to a concentration value equal to the solute concentration in the soil. However, we can observe some differences in the early dynamics of the solute uptake process. Diffusive uptake with large permeability reach an equilibrium between root and soil at the first time step. As a consequence, the root concentration reaches its plateau earlier than under advective uptake (Figure S2a).

Figure S2b shows the solute collar concentration, i.e. the mass of solute reaching the collar divided by the volume of water transpired. Under the solute uptake properties of these scenarios, we expect solute collar concentrations that are equal to the concentration in the soil. The results from the fully advective scenario show that solute collar concentration reaches the plateau at less than 1 day after the start of the simulation. Simulations with P=100 cm d^{-1} reach a plateau

earlier due the fast equilibrium between soil and root. Once the equilibrium is reached, collar concentrations slowly increase towards the concentration in the soil.

The comparison between the simulations with normal exchange and equilibrium approaches showed a good agreement between both methods and with the fully advective uptake scenario (Figure S3). The cumulative mass reaching the root collar was the same for all simulations (Figure S3a). In addition, the collar solute concentration in all simulations reached an equilibrium at about 2 10^{-7} g cm^{-3}, which corresponded with the sink-averaged concentration in the soil (Figure S3b). These results demonstrate that the newly implemented equilibrium approach is able to describe fast diffusive uptake dynamics while keeping a reasonable time step size.

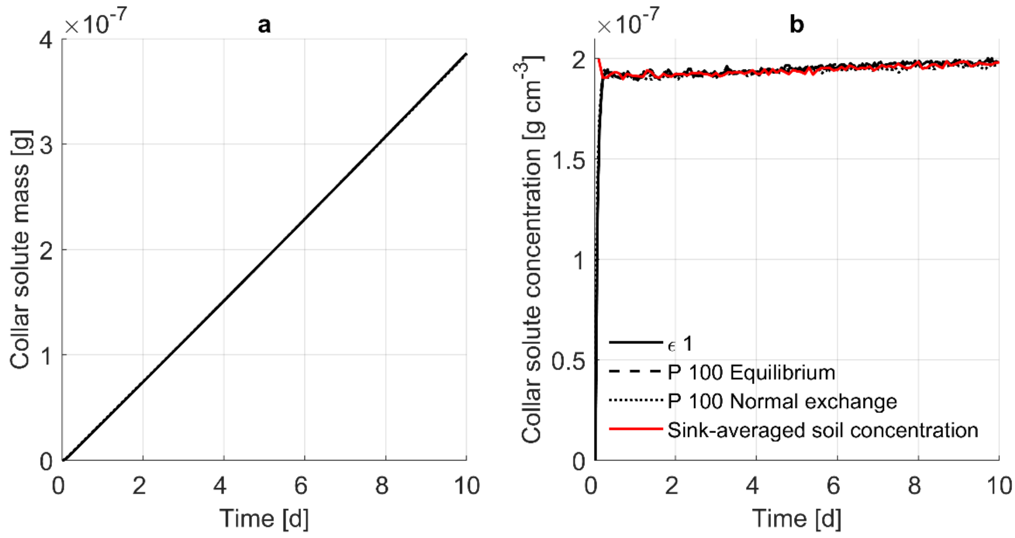

Figure S3. Comparison between simulations with fully advective uptake (ε = 1, solid line) and fully diffusive uptake with large P (P = 100 cm d^{-1}) with the equilibrium approach (dt = 0.01 days, dashed line) and the normal exchange approach with smaller time steps (dt = 0.0001 days, dotted line). Collar solute concentration (b) is the solute mass that reaches the collar divided by the amount of transpired water at each time step. The sink-averaged concentration was calculated from the fully advective scenario. The results correspond to simulations with dx=0.3 cm.

C. Additional information on FOCUS Hamburg scenario

Table S2. Depth dependent correction factor for the degradation rate in the FOCUS model PEARL.

Soil depth (cm)	f_d [-]
0 – 30	1.0
30 – 60	0.5
60 – 100	0.3
> 100	0

Table S3. Soil hydraulic properties for the Hamburg scenario. θs saturated water content; θr residual water content; α, n, m, λ van Genuchten shape parameters; K_{sat} saturated hydraulic conductivity, ρ_b is the soil bulk density, OM is the organic matter content and K_F is the Freundlich sorption coefficient, om is the organic matter in the respective soil layer.

Depth	θ_s	θ_r	α	n	m	K_{sat}	λ	ρ_b	OM	K_F*
cm	$m^3\ m^{-3}$	$m^3\ m^{-3}$	m^{-1}	-	-	$x10^{-6}\ m\ s^{-1}$	-	$g\ cm^{-3}$	$g\ g^{-1}$	$cm^3\ g^{-1}(g\ cm^{-3})^{(1-n)}$
0-30	0.391	0.036	1.491	1.468	0.3188	23.33	0.5	1.5	0.026	0.064
30-60	0.370	0.030	1.255	1.565	0.3610	31.67	0.5	1.6	0.017	0.043
60-75	0.351	0.029	1.808	1.598	0.3742	28.33	0.5	1.56	0.0034	0.00854
75-90	0.310	0.015	2.812	1.606	0.3773	28.33	0.5	1.62	0	0.0
90-145	0.310	0.015	2.812	1.606	0.3773	28.33	0.5	1.6	0	0.0

* Freundlich equilibrium sorption is assumed in the soil domain and sorption parameter $K_{F,S}$ is derived for each soil layer by $K_{F,S} = K_{OM} * f_{OM}$ (Bromilow and Chamberlain, 1995), where K_{OM} is the solute sorption parameter and f_{OM} the fraction of organic matter in the respective soil.

Table S4. Selected properties of dummy substance B in the FOUCS scenarios.

Property	Symbol	Unit	
Molecular weight	M	$g\ mol^{-1}$	300
Vapour pressure	Vp	mPa	0.1
Solubility in water	S_W	$mg\ L^{-1}$	90
Reference degradation rate	$k_{S,r}$ (T = 293 K $\theta_s = \theta_{fc}$)	d^{-1}	0.03
Sorption parameter organic matter	K_{OM}	$cm^3\ g^{-1}$	10
Sorption parameter organic carbon	K_{OC}	$cm^3\ g^{-1}$	17
Freundlich isotherm exponent	n	-	0.90
Diffusion coefficent in water	D_W	$m^2\ d^{-1}$	$4.30\ 10^{-5}$

Metabolites		-	No
Lipophilicity*	$\log K_{OW}$	-	1.41
Root membrane permeability**	P_M	cm d^{-1}	13.44
Root sorption coefficient***	$K_{D,R}$	cm^3 g^{-1}	0
Plant Uptake Factor	PUF	-	0.5
Application rate		kg $ha^{-1}year^{-1}$	1
Date of application		-	1 day before emergence
Incorporation depth		cm	0

* (Seth et al., 1999), **(Grayson and Kleier, 1990), ***negligible for logKow<2 (Trapp, 2000).

Table S5. Root properties

Property	Symbol	Unit	
Root water content*	θ_R	-	0.94
Root dry bulk density*	ρ_R	g cm^{-3}	1

*(Trapp, 2000).

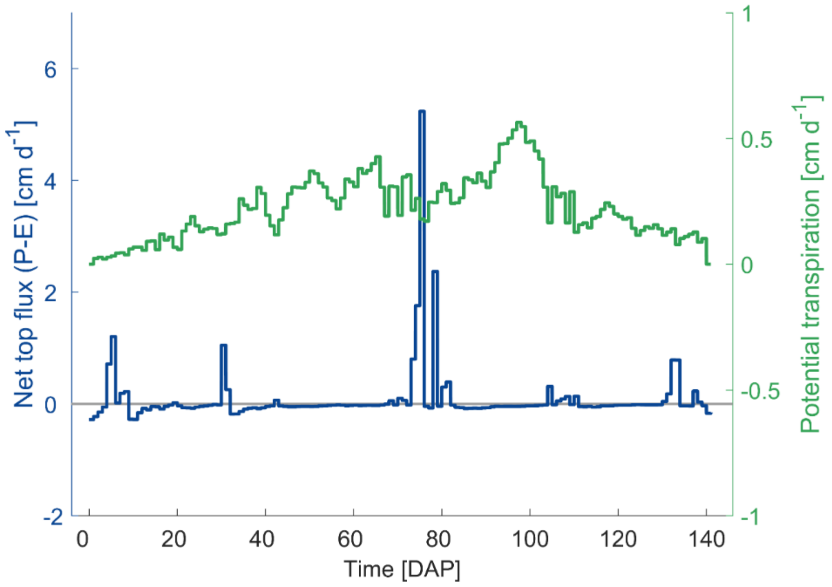

Figure S4: Climatic input data for the 142 day-long vegetation period. Net water flux at the soil surface (precipitation – evaporation) [cm day^{-1}] is plotted in blue; potential transpiration flux [cm day^{-1}] is plotted in green.

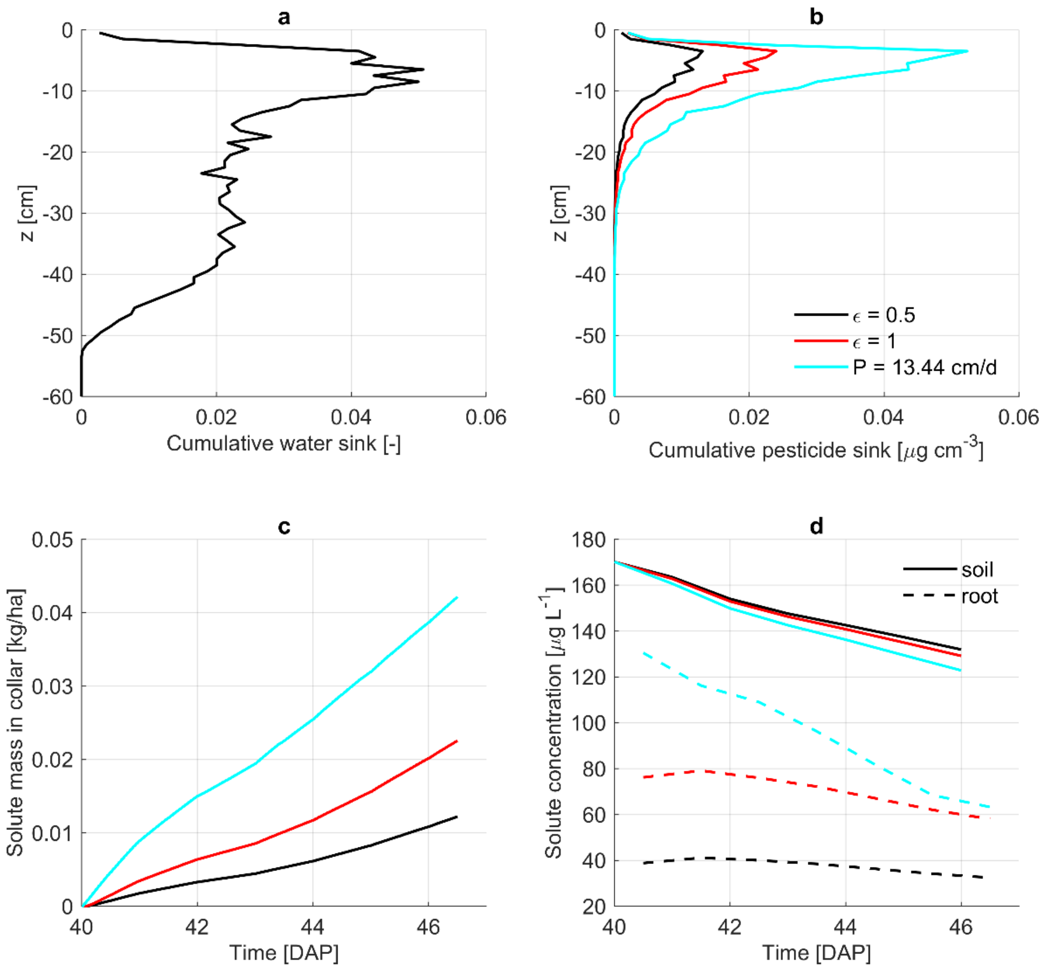

Figure S5. Cumulative water (a) and solute (b) sink distributions, cumulative uptake (c) and daily average root and soil concentration (d) results for the 1-week simulations performed with a complex maize root architecture. Root solute uptake is modelled as an advective mechanism with ε = 0.5 (black) and ε = 1 (red), and as a diffusive mechanism with P = 13.4 cm d^{-1} (cyan).

References

Bechtold, M., Vanderborght, J., Ippisch, O., Vereecken, H., 2011. Efficient random walk particle tracking algorithm for advective-dispersive transport in media with discontinuous dispersion coefficients and water contents: Efficient random walk particle tracking algorithm. Water Resour. Res. 47, n/a-n/a. https://doi.org/10.1029/2010WR010267

Boesten, J., Businelli, M., Delmas, A., Gottesbüren, B., Hanze, K., Jarvis, T., Jones, R., Klein, M., Roquero, C., Maier, W.-M., Styczen, M., Thorsen, M., Travis, K., Vanclooster, M., 2000. FOCUS groundwater scenarios in the EU review of active substances 122.

Bromilow, R.H., Chamberlain, K., 1995. Principles governing uptake and transport of chemicals (No. BIOSIS/95/17913). Bromilow, RH; Chamberlain, K.

Grayson, B.T., Kleier, D.A., 1990. Phloem mobility of xenobiotics. IV. Modelling of pesticide movement in plants. Pestic. Sci. 30, 67–79. https://doi.org/10.1002/ps.2780300108

Huber, K., Vanderborght, J., Javaux, M., Schröder, N., Dodd, IanC., Vereecken, H., 2014. Modelling the impact of heterogeneous rootzone water distribution on the regulation of transpiration by hormone transport and/or hydraulic pressures. Plant Soil 1–20. https://doi.org/10.1007/s11104-014-2188-4

Jacques, D., Šimůnek, J., Mallants, D., van Genuchten, M.Th., 2006. Operator-splitting errors in coupled reactive transport codes for transient variably saturated flow and contaminant transport in layered soil profiles. J. Contam. Hydrol. 88, 197–218. https://doi.org/10.1016/j.jconhyd.2006.06.008

Schröder, N., Javaux, M., Vanderborght, J., Steffen, B., Vereecken, H., 2012. Effect of root water and solute uptake on apparent soil dispersivity: A simulation study. Vadose Zone J. 11, 0. https://doi.org/10.2136/vzj2012.0009

Schröder, N., Lazarovitch, N., Vanderborght, J., Vereecken, H., Javaux, M., 2013. Linking transpiration reduction to rhizosphere salinity using a 3D coupled soil-plant model. Plant Soil. https://doi.org/10.1007/s11104-013-1990-8

Seth, R., Mackay, D., Muncke, J., 1999. Estimating the Organic Carbon Partition Coefficient and Its Variability for Hydrophobic Chemicals. Environ. Sci. Technol. 33, 2390–2394. https://doi.org/10.1021/es980893j

Trapp, S., 2000. Modelling uptake into roots and subsequent translocation of neutral and ionisable organic compounds. Pest Manag Sci 12.
